# A Machine Learning Approach to Passively Informed Prediction of Mental Health Risk in People with Diabetes: Retrospective Case-Control Analysis

**DOI:** 10.2196/27709

**Published:** 2021-08-27

**Authors:** Jessica Yu, Carter Chiu, Yajuan Wang, Eldin Dzubur, Wei Lu, Julia Hoffman

**Affiliations:** 1 Livongo Health, Inc Mountain View, CA United States

**Keywords:** diabetes mellitus, mental health, risk detection, passive sensing, ecological momentary assessment, machine learning

## Abstract

**Background:**

Proactive detection of mental health needs among people with diabetes mellitus could facilitate early intervention, improve overall health and quality of life, and reduce individual and societal health and economic burdens. Passive sensing and ecological momentary assessment are relatively newer methods that may be leveraged for such proactive detection.

**Objective:**

The primary aim of this study was to conceptualize, develop, and evaluate a novel machine learning approach for predicting mental health risk in people with diabetes mellitus.

**Methods:**

A retrospective study was designed to develop and evaluate a machine learning model, utilizing data collected from 142,432 individuals with diabetes enrolled in the Livongo for Diabetes program. First, participants’ mental health statuses were verified using prescription and medical and pharmacy claims data. Next, four categories of passive sensing signals were extracted from the participants’ behavior in the program, including demographics and glucometer, coaching, and event data. Data sets were then assembled to create participant-period instances, and descriptive analyses were conducted to understand the correlation between mental health status and passive sensing signals. Passive sensing signals were then entered into the model to train and test its performance. The model was evaluated based on seven measures: sensitivity, specificity, precision, area under the curve, F_1_ score, accuracy, and confusion matrix. SHapley Additive exPlanations (SHAP) values were computed to determine the importance of individual signals.

**Results:**

In the training (and validation) and three subsequent test sets, the model achieved a confidence score greater than 0.5 for sensitivity, specificity, area under the curve, and accuracy. Signals identified as important by SHAP values included demographics such as race and gender, participant’s emotional state during blood glucose checks, time of day of blood glucose checks, blood glucose values, and interaction with the Livongo mobile app and web platform.

**Conclusions:**

Results of this study demonstrate the utility of a passively informed mental health risk algorithm and invite further exploration to identify additional signals and determine when and where such algorithms should be deployed.

## Introduction

In the United States, 34.2 million people are affected by diabetes mellitus [1]. Approximately 25% of those living with diabetes experience significant depressive symptoms, and up to 40% experience generalized anxiety disorder (GAD) [1-3]. Individuals with diabetes and mental health challenges have been found to be less adherent to diabetes treatment recommendations, including diet, exercise, medication use, glucose monitoring, and medical appointments, and they are at a greater risk for adverse medical outcomes [1]. Health care costs for those with comorbid diabetes and mental health have been estimated to be US $4 billion to $9 billion greater than for those without these conditions [4]. However, proactive detection of mental health needs of people with diabetes could facilitate early intervention, thereby improving their overall health and quality of life and reducing the health and economic burdens placed on this population and the health care system as a whole. 

Despite recommendations by the American Diabetes Association and the United States Preventive Services Task Force to routinely evaluate people with diabetes for their mental health needs, only 25% to 50% of people with diabetes who have depression receive a mental health diagnosis and intervention [5,6]. This gap in receiving care is a result of a shortage of mental health professionals available to offer assessment and intervention, a lack of mental health knowledge among primary care providers who most often care for patients with diabetes, and limited access to mental health screening tools in health care practices offering services to these patients [6]. Newer methods such as passive sensing and ecological momentary assessments (EMAs) provide a more scalable, less effort- and time-intensive approach to information gathering and assessment. Passive sensing refers to the capture of data about a person without any extra effort on their part [7]. EMA refers to the repeated sampling of an individual’s behavior in real time within their natural environment [8]. Both methods can be integrated into or with devices and services that people with diabetes already utilize in their daily lives, such as blood glucose meters, smartphones, and health coaching platforms to enable the collection and processing of data in real time and to provide context for real-time interventions [7]. 

Although passive sensing and EMA have previously been examined in the general population, limited studies have focused on the detection of mental health needs among the diabetes population outside of using smartphones as data warehouses, relying on accelerometer, GPS, ambient light sensors, and call log data [9,10]. Moreover, no known study to date has attempted to detect mental health concerns in people with diabetes by using blood glucose meters despite the fact that individuals living with diabetes are encouraged to engage with these devices at regular intervals, blood glucose monitoring has been found to be correlated with psychological effects, and engagement with these devices and testing blood glucose levels are known to be associated with mood or stress [11-13]. Further, blood glucose meter data can be paired with data from other sources for a robust view of a person’s behavioral and emotional profile. The primary aim of this study was to conceptualize, develop, and evaluate a novel approach using passive sensing for predicting mental health risk in people with diabetes.

## Methods

### Study Design

A multidisciplinary team of experts in data science, machine learning, and clinical and experimental psychology collaborated in the development of a machine learning model for detecting potential mental health risk from passive sensing signals that was both clinically relevant and statistically rigorous. A retrospective analysis was performed to evaluate the machine learning model for detecting potential mental health risk from passive sensing signals leveraging data collected during participants’ engagement in the Livongo for Diabetes program [14].

### Livongo for Diabetes

The Livongo for Diabetes program is a digital remote program for the management of chronic condition focused on empowering members by providing education and tools to self-manage their diabetes through mobile technology. The program offers members (1) a cellular-enabled, two-way messaging device that measures blood glucose and delivers personalized insights; (2) free, unlimited blood glucose test strips; (3) real-time support from diabetes response specialists available 24 hours a day, 7 days a week, 365 days a year; and (4) access to certified diabetes care and education specialists for support and goal setting. Further details on the Livongo for Diabetes program and its efficacy in improving diabetes-related outcomes are available in the literature [15-17]. 

### Study Participants

Study participants were defined as those individuals enrolled in the Livongo for Diabetes Program between January 1, 2018, and February 28, 2020, who used their blood glucose meter at least once (N=142,432). Approval was granted by the Aspire Independent Review Board (#520160099). All participants provided consent to participate, and guidelines outlined in the Declaration of Helsinki were followed. 

### Study Procedure

The mental health status of each participant was verified through available data from two sources that included data on medications prescribed to and filled by participants and mental health–related interventions. Next, passive sensing signals were extracted from participants’ behaviors as they interacted with Livongo’s blood glucose meter, mobile app, web portal, and coaching feature. Then, data sets were assembled by aggregating these signals per participant over various periods, creating *participant-period* instances. Descriptive analyses were conducted to understand the correlation between the signals and mental health status. Finally, demographic information and passive sensing signals were entered into the model, training it to understand the relationships between these signals and the participants’ mental health status.

### Study Population Identification

Identification of population *cases* and *controls* with respect to mental health conditions—which, in the context of labeling data for model training, we refer to as *ground truth*—was performed utilizing two data sources: (1) claims data and (2) medication prescription data. Claims data contained information on medications indicated for mental health conditions that were prescribed to and filled by participants, as well as mental health–related assessments and interventions. Based on data availability and right to use, 6.1% (8633/142,432) of the study participants had claims data, which provided a diverse way to identify their mental health needs through diagnoses, procedures, and prescriptions. Medication prescription data contained only information on mental health–related medications that were prescribed to and filled by participants. Medication prescription data were available for the entire study population and used to identify cases for the remaining participants. Participant demographic characteristics, which were used to evaluate signals correlated with mental health in the study population, are summarized in Table 1.

**Table 1:** Participant demographics and characteristics at the time of enrollment (N=142,432).

Characteristic			Value
Age in years, mean (SD)			54.8 (12.4)
**Gender, n (%)**			
	Female	68,968 (48.4)	
	Male	73,147 (51.6)	
	Other	317 (0.22)	
**Ethnicity, n (%)**			
	Hispanic	12,809 (9.0)	
	Non-Hispanic	86,116 (60.5)	
	Unknown	43,507 (30.6)	
**Race, n (%)**			
	Caucasian	66,551 (46.7)	
	Black or African American	14,702 (10.3)	
	Asian	8199 (5.76)	
	Pacific Islander	468 (0.33)	
	American Indian	725 (0.51)	
	Other	6,588 (4.63)	
	Unknown	45,199 (31.7)	
**Diabetes type, n (%)**			
	Type 1	14,360 (10.1)	
	Type 2	126,369 (88.7)	
	Unknown	1603 (1.2)	
Years since diagnosis, mean (SD)			8.27 (8.1)
First reported A_1c_, mean (SD)			7.51 (1.7)
**Insulin use, n (%)**			
	Yes	39,153 (27.5)	
	No	102,622 (72.1)	
	Unknown	657 (0.5)	

### Passive Sensing Signals

All data utilized in the study were collected in the course of how participants naturally engaged with the Livongo for Diabetes Program. That is, no data were collected solely for study purposes. We identified various data sources potentially useful to detect mental health risk behaviors. From these data sources, we extracted 83 individual signals that can be broadly classified into the following four categories. Note that individual signal names are withheld to protect proprietary information.

#### Demographics

Demographic factors such as age, gender, ethnicity, and race have been shown to be related to mental health [18]. Therefore, we included participants’ demographic data into the model.

#### Glucometer Data

The Livongo blood glucose meter is the most frequent interaction point for participants of the Livongo for Diabetes Program. Low rates of blood glucose monitoring [2] and poorer blood glucose control [19] have been linked to depression among those with diabetes; and depression, anxiety, and stress symptoms are greater among people with diabetes than those without [20]. Differences in device usage is particularly informative of conditions such as depression when examining usage time of day [10] and weekday [21]. Therefore, the key metrics derived from glucometer usage included the number of times blood glucose was checked; blood glucose levels; and variations, responses to questions to assess context such as current emotional state, and time of the day and day of week when the reading was taken. 

#### Coaching Data

In the Livongo for Diabetes Program, Livongo coaches contact individuals under certain conditions. Numerous studies have affirmed relationships between sociability and mental health. Fewer calls and fewer incoming texts have been linked to depression [22], whereas frequency and duration of conversations have been shown to be useful in evaluation of bipolar disorder [23]. Coaching data can serve as a proxy for sociability, for which successful or failed contacts and time spent interacting can be used to glean valuable insights.

#### Event Data

In addition to the blood glucose meter, individuals enrolled in the Livongo for Diabetes Program interact with multiple platforms during program participation, including the Livongo mobile app and web portal. In following the motivations behind utilizing glucometer and coaching data, we collected frequency, duration, interactivity, and consistency of interaction sessions, as well as the time of day and day of week information associated with the use of the mobile app and web portal. In addition, we tracked voluntary report sharing with friends and family as well as interactions with pop-up reminders.

### Statistical Analyses

We conducted correlation analyses using Pearson *r* to preliminarily gauge the strength of the relationship between each extracted signal and the presence of mental health conditions. Among the most highly correlated signals, demographics were well represented, including gender (male: *r=*–0.156, *P*<.001; female: *r*=0.155, *P*<.001), race (Asian: *r*=–0.104, *P*<.001; White: *r*=0.101, *P*<.001; Black: *r*=–0.041, *P*<001), BMI (*r*=0.086, *P*<.001), and smoking status (active smoker: *r*=0.047, *P*<.001). With regard to glucometer, coaching, and event data, responses on current emotional state from participants during blood glucose checks indicating wellness (*r*=–0.108, *P*<.001) or unwellness (*r*=0.121, *P*<.001) were most strongly correlated. Greater frequency and consistency of interactions with services were negatively associated with mental health conditions. In addition, we found that greater variation in blood glucose values, as measured by SD, were positively correlated with mental health conditions (*r*=0.041, *P*<.001).

### Outcome Data

When quantifying the performance of a prospective model for identifying mental health risk, it is important to consider a variety of perspectives. Metrics such as total accuracy are inadequate if used alone because they can hide model deficiencies on imbalanced data. Consider these three questions, which cannot be answered with total accuracy alone but are of particular importance in a diagnostic setting: 

1. How often are those with mental health needs (cases) correctly identified?
2. How often are those without mental health needs (controls) correctly identified? 
3. How often are those with predicted mental health needs truly cases?

The following seven measures commonly used in machine learning model evaluation [24] were selected to address the above questions and beyond, enabling a holistic view of model performance:

1. Sensitivity or recall, which addresses question 1
2. Specificity, which addresses question 2
3. Precision, which addresses question 3
4. Area under the curve (AUC), defined as the area under the receiver operating characteristic curve—an important measure quantifying the model’s capacity to differentiate *cases* and *controls* (1=ideal performance, 0.5=random prediction)
5. F_1_ score, defined as the harmonic mean of the precision and recall
6. Accuracy, defined as the proportion of instances correctly classified
7. The confusion matrix, which depicts the number of correctly and incorrectly identified cases and controls

### Model Development

To develop our machine learning model, we then had to divide the study population into two segments. The first segment, termed the training and validation set, enabled the model to learn. The second segment, termed the test sets, was held separate from model training and used to evaluate the model’s ability to generalize to unseen data. For training, we used a time-interval slice of the population consisting of 124,322 participants (ie, 87% of the study population) who had activated their blood glucose meters in 2018 or 2019, with passive sensing signal data collected in that timeframe. For testing, we defined three distinct test sets designed to comprehensively evaluate model performance. The first two test sets used medication prescription or refill data as their sources of ground truth, whereas the third test set used medical and pharmacy claims data as its source of ground truth.

1. The first test set (test set 1) consisted of the same participant subset as the training data, but with signal data collected in the first two months of 2020. This data subset evaluated model prediction capability on previously seen participants (93,155/142,432, 65.4%). 
2. The second test set (test set 2) consisted of participants activated in the first two months of 2020 and the associated signal data. This test set evaluated model prediction capability on new, unseen participants (9477/142,432, 6.7%).
3. The third test set (test set 3) utilized the claims data by identifying participants activated in 2018, 2019, or the first two months of 2020. This final data subset evaluated prediction capability with regard to unseen participants, with mental health needs identified through more diverse sources beyond prescriptions only (8633/142,432, 6.1%).

**Figure 1:**
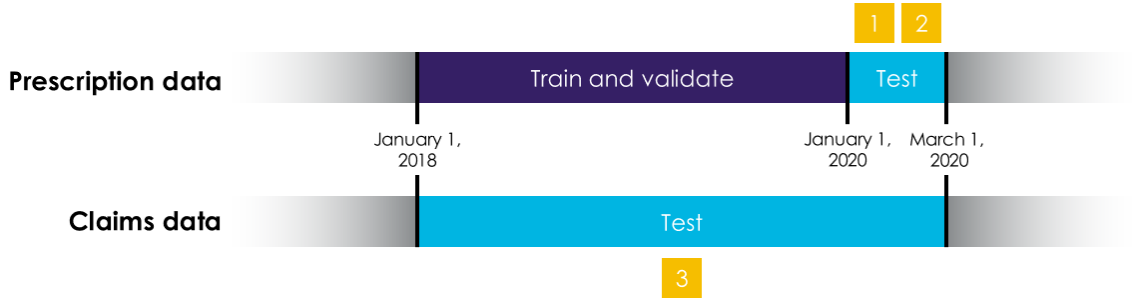
Source and date ranges of data subsets defined in this study.

A visual summary of the data subsets is presented in Figure 1, and the specific numeric breakdowns are described in Table 2. Furthermore, the demographic information for each subset is detailed in Multimedia Appendix 1.

To increase the model utility, passive signals were aggregated during a certain period (participant-period) and presented to the model for prediction of mental health risk. We defined the participant-period as an instance. In this study, an aggregation window of 4 weeks was selected to optimize data availability. Furthermore, two additional conditions were applied to filter out ineligible participant-period instances: (1) instances before a participant had participated in the Livongo Program and (2) instances of extended inactivity, defined as 30 or more days without any interaction with the Livongo for Diabetes Program.

The rationale for the first criterion is trivial. The second eligibility condition reflects the reasoning that a model for identifying mental health needs from passive signals should only be employed when a signal is present, specifically signals where missingness cannot be assumed to be zero (eg, blood glucose values). Thus, our model should only be trained and evaluated on complete instances. Table 3 demonstrates the distribution of eligible instances among the data subsets. Note the class imbalance, with control instances represented at roughly a 2:1 ratio over case instances in each data subset.

**Table 2:** Number of participant cases and controls for each data subset.

Data subset	Cases, n (%)	Controls, n (%)	Subset total, n
Training or validation set	42,481 (34.2)	81,841 (65.8)	124,322
Test set 1	31,251 (33.5)	61,904 (66.5)	93,155
Test set 2	2919 (30.8)	6558 (69.2)	9477
Test set 3	3358 (38.9)	5275 (61.1)	8633
Total unique	48,758 (34.2)	93,674 (65.8)	142,432

**Table 3:** Number of participant-period cases and controls for each data subset.

Data subset	Cases, n (%)	Controls, n (%)	Subset total, n
Training and validation set	287,311 (34.3)	549,183 (65.7)	836,494
Test set 1	54,709 (33.3)	109,550 (66.7)	164,259
Test set 2	2953 (30.8)	6640 (69.2)	9593
Test set 3	34,190 (40.1)	51,150 (59.9)	85,340
Total	379,163 (34.6)	716,523 (65.4)	1,095,686

A machine learning model was enlisted to capture the relationship between input activity features and exhibited mental health needs. The core component of our approach was the training of LightGBM [25] gradient tree boosting models on random subsets of the training data. This approach addressed the class imbalance; we undersampled the training control instances by random undersampling, thus reducing the number of control instances to equal the number of case instances. Because this technique reduced the number of control instances by roughly half, we saw the opportunity to train multiple models on multiple random subsets. This strategy enabled us to fully utilize the entire training data set, with each model training on a differing perspective of the data. We utilized soft voting to obtain an output prediction for a given instance, meaning the outputs of each constituent model—a value from 0 to 1 interpretable as the confidence that an instance is a case—were averaged to obtain a single aggregate confidence score. 

Our final devised model consists of an ensemble of 10 LightGBM models. During our model selection process, we evaluated multiple other classes of machine learning models, including logistic regression, random forests, and neural networks. We also experimented with other flavors of gradient boosting, including XGBoost and CatBoost, and overall, LightGBM yielded the highest performance for our training task. To tune each constituent model, we used automated hyperparameter tuning enabled by the hyperopt [26] Python library with 5-fold cross-validation on the training set. Following this training procedure, we evaluated the model on the three held-out test sets to assess model performance.

## Results

**Figure 2:**
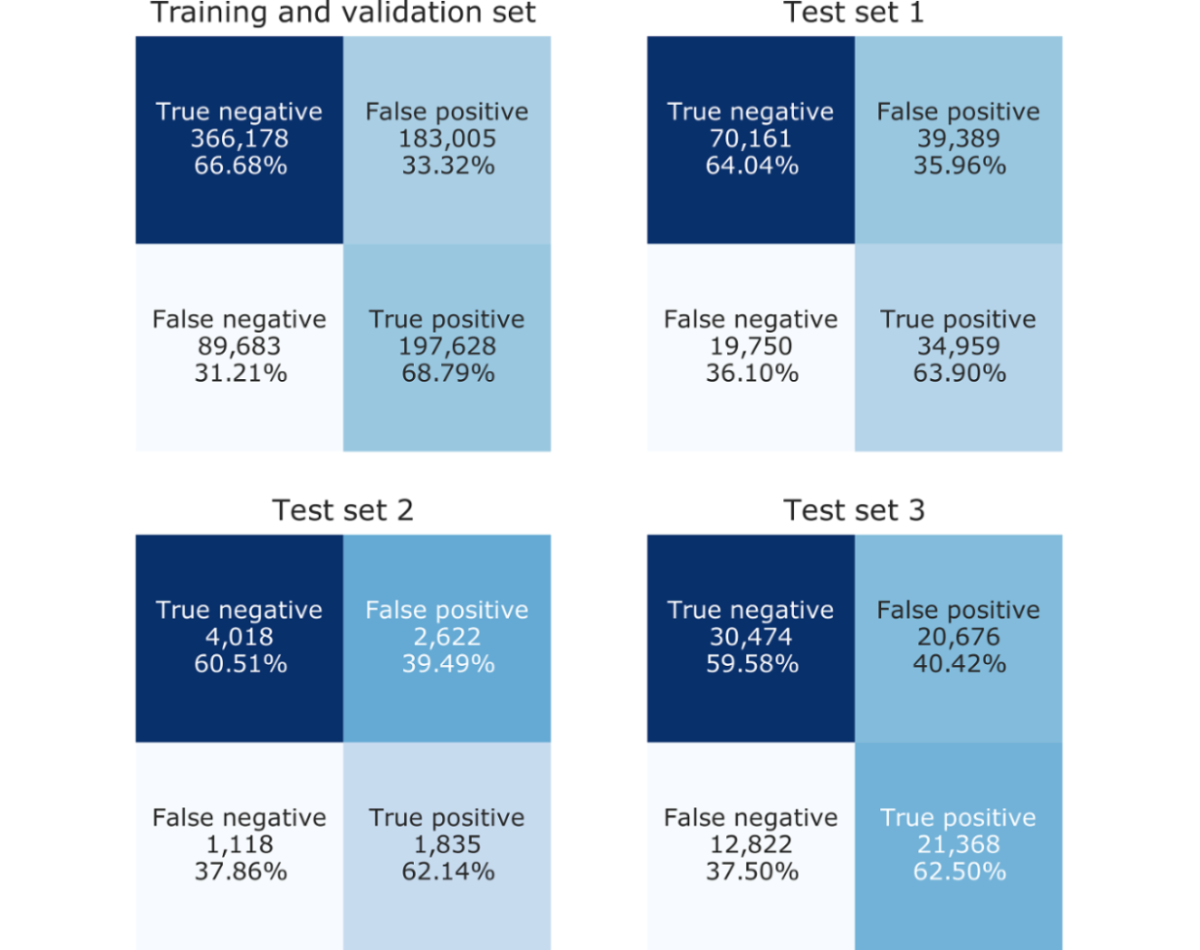
Confusion matrices for each data subset.

The results of our model’s performance on each of the previously described data subsets are presented in Table 4. In addition, the associated confusion matrices are shown in Figure 2, with both counts and percentages (normalized by class support size) depicted. Across all three test sets, the vast majority of metrics exceeded 0.5. Notably, we achieved an AUC of nearly 0.7 on the first test set and exceeded 0.65 across all three sets. The metrics for which the model fell short of the 0.5 mark were precision in the first and second test sets and the F_1_ score for the second test set. However, it is important to note that owing to class imbalance, 0.5 would not be the theoretical precision or the F_1_ score yielded by random prediction. Rather, the precision obtained by random prediction would be the proportion of cases, with the F_1_ score affected commensurately. In our case, baseline precision would be approximately 0.3331 for the first test set and 0.3078 for the second, both of which were well outperformed by the reported precisions of 0.4702 and 0.4164. Likewise, the baseline theoretical F_1_ score of 0.3810 was greatly outperformed by the reported 0.4953 on the second test set. Concerning improvement over random prediction overall, our model produced an approximately 14-point gain in precision for the first test set and an approximately 10-point gain for the second and third sets, whereas recall improved by 14 and 12 points and AUC improved by 20 and 16 points, respectively. These results demonstrate a respectable, generalizable performance and are an encouraging advancement towards practical passive mental health risk assessment at scale.

**Table 4:** Performance metrics for each data subset.

Data subset	Sensitivity	Specificity	Precision	AUC^a^	F_1_ Score	Accuracy
Training and validation set	0.688	0.667	0.519	0.745	0.592	0.674
Test set 1	0.639	0.640	0.470	0.696	0.542	0.640
Test set 2	0.621	0.605	0.412	0.658	0.495	0.610
Test set 3	0.625	0.596	0.508	0.656	0.561	0.608

^a^AUC: area under the curve.

Our model results also provided the opportunity to gain further insight into the utility of the passive sensing signals. For this purpose, we used the SHapley Additive exPlanations (SHAP) method [27] to compute feature importance, allowing us to quantify the average contribution of each signal to the model. These values are presented in Table 5. Our findings closely mirrored the insights from our correlation analysis. Demographics ranked highly among signals, with gender and race identified as the two most relevant factors. Among passive sensing signals, responses indicating an emotional state of wellness or unwellness during blood glucose checks were deemed the most important. Interaction frequency and consistency were also considered valuable according to SHAP. The model took into particular consideration blood glucose checks based on the times of day and the proportion of days they were performed. Mean and SD values of blood glucose levels also appeared in the top quartile of signals.

**Table 5:** Feature importance as measured by mean absolute SHapley Additive exPlanations (SHAP) values, interpreted as the average impact on model output (log-odds) magnitude. Note that aliases based on the signal category are depicted in lieu of the signals to protect proprietary information.

Passive signal	Mean absolute SHAP value
Demographics 1	0.152
Demographics 2	0.089
Glucometer data 1	0.078
Demographics 3	0.040
Glucometer data 2	0.039
Demographics 4	0.037
Demographics 5	0.037
Event data 1	0.022
Demographics 6	0.018
Event data 2	0.017
Glucometer data 3	0.014
Glucometer data 4	0.013
Glucometer data 5	0.012
Glucometer data 6	0.012
Glucometer data 7	0.011
Demographics 7	0.011
Glucometer data 8	0.011
Glucometer data 9	0.010
Glucometer data 10	0.008
Glucometer data 11	0.008
Glucometer data 12	0.008
Glucometer data 13	0.007
Event data 3	0.006
Event data 4	0.006
Glucometer data 14	0.006
Glucometer data 15	0.006
Event data 5	0.006
Event data 6	0.005
Glucometer data 16	0.005
Event data 7	0.005
Event data 8	0.004
Event data 9	0.004
Event data 10	0.004
Glucometer data 17	0.004
Event data 11	0.003
Glucometer data 18	0.003
Coaching data 1	0.003
Glucometer data 19	0.002
Event data 12	0.002
Event data 13	0.002
Coaching data 2	0.002
Event data 14	0.002
Event data 15	0.002
Glucometer data 20	0.002
Event data 16	0.002
Coaching data 3	0.001
Event data 17	0.001
Event data 18	0.001
Coaching data 4	0.001
Event data 19	0.001
Event data 20	<0.001
Event data 21	<0.001
Event data 22	<0.001
Event data 23	<0.001
Coaching data 5	<0.001
Event data 24	<0.001
Event data 25	<0.001
Event data 26	<0.001
Event data 27	<0.001
Event data 28	<0.001
Coaching data 6	<0.001
Coaching data 7	<0.001
Event data 29	<0.001
Event data 30	<0.001
Event data 31	<0.001
Event data 32	<0.001
Event data 33	<0.001
Event data 34	<0.001
Event data 35	<0.0001
Event data 36	<0.0001
Event data 37	<0.0001
Event data 38	<0.0001
Event data 39	<0.0001
Event data 40	<0.0001
Event data 41	<0.0001
Event data 42	<0.0001
Event data 43	<0.0001
Event data 44	<0.0001
Coaching data 8	<0.0001
Coaching data 9	0
Coaching data 10	0
Coaching data 11	0
Event data 45	0

## Discussion

In this study, we found that a machine learning approach using passive sensing signals that included data on participant demographics, blood glucose meter use, interaction with diabetes coaches as a proxy for sociability, and engagement with the Livongo for Diabetes Program demonstrated utility in predicting mental health risk among people with diabetes.

The results of our approach invite further exploration and expansion. It is well understood that smartphones can be viewed as vehicles for passive data collection and help identify digital phenotypes of mental health disorders, as shown previously [28]. However, it is time to move beyond focusing on smartphones as the only devices that enable passive sensing and EMA and view other devices and services that people with diabetes must use for their self-management as robust data warehouses. In this particular study, participants who enrolled in the Livongo for Diabetes Program had access to a Bluetooth-enabled blood glucose meter for measuring their blood glucose levels, the Livongo mobile app and web platform for tracking food intake and physical activity as well as receiving health reminders, and Livongo coaches for coaching for diabetes self-management. Each device and service offered valuable data to input in our model. The blood glucose meters provided access to the participants’ behavioral, emotional, and physiological data, such as how they were feeling at the time of measuring their blood glucose level and the reading itself. The Livongo mobile app and web platform enabled us to understand when participants were awake, using their smartphones, and engaged in a health-related activity. Coaching allowed us to understand whether participants were actively communicating with others. Together, these different data sources enabled us to create a data set that combined behavioral, emotional, and physiological factors into a holistic predictive algorithm. Although these particular devices and services are unique to Livongo members, there are ways to obtain similar data in the real world. For example, several commercially available wireless and Bluetooth-enabled blood glucose meters connect to mobile apps that enable people with diabetes mellitus to track and receive feedback on their blood glucose levels and share data with others. Such meters and associated apps host behavioral, communication, and physiological data similar to what we used in our model. There is also a plethora of health-related apps that enable individuals to track their food intake, physical activity, mood, sleep, and other health signals. These apps host additional behavioral and emotional data similar to what we used in our model.

Identifying potential mental health risk from passively collected signals is undoubtedly not a simple task, and our study has some limitations. First, because we limited our extracted signals to interactions with Livongo devices and applications, we did not have access to certain passive signals shown to be predictive in previous studies, such as mobile device accelerometer, ambient light sensor, or GPS data. As a result, our model was given a somewhat restricted view of a member’s activity and sociability patterns. Second, we had access to a limited volume of medical and pharmacy claims data, which made it difficult to utilize the data on their own. However, both limitations could also be seen as strengths of our study. Our inability to access previously studied passive signals afforded us an opportunity to examine new signals from devices and services that are unique to people with diabetes. It may also be more acceptable from a privacy perspective. Furthermore, we had limited claims data; nevertheless, the data enabled us to confidently label participants as *cases* versus *controls*. Finally, a major strength of our study was the fact that, by design, no data were collected with active participant input for the express purpose of detecting mental health risk. In that regard, our proposed approach makes real-world deployment more readily feasible, in contrast to other studies of passive sensing and EMA for mental health, which required active participant participation and extensive sensor infrastructure.

In sum, our model is a bold step toward detecting potential mental health risk passively and autonomously. In its nascent stage, we recommend integrating such a model with existing systems and services, while continuing to improve the quality and completeness of care that can be offered to those dealing with mental health needs.

## Appendix

Multimedia Appendix 1 Demographics distribution across various data sets.

## Abbreviations

AUC: area under the curve
EMA: ecological momentary assessment
GAD: generalized anxiety disorder
SHAP: SHapley Additive exPlanations
