# Supplementary material for: A Machine Learning Approach to Passively Informed Prediction of Mental Health Risk in People with Diabetes: Retrospective Case-Control Analysis
Source: J Med Internet Res. 2021 Aug 27;23(8):e27709. doi: 10.2196/27709 (PMC8433872; doi:10.2196/27709)
Supplement: Multimedia Appendix 1 [file jmir_v23i8e27709_app1.docx]

**Appendix A: Demographics Distribution**

| **Training Set** | | |  |  |  |  |  |  |  |  |
| --- | --- | --- | --- | --- | --- | --- | --- | --- | --- | --- |
| **Gender** | **Case** | **Control** |  | **Race** | **Case** | **Control** |  | **Age** | **Case** | **Control** |
| **Female** | 25131 | 34907 |  | **American Indian** | 230 | 397 |  | **Mean** | 55.16 | 54.44 |
| **Male** | 17242 | 46734 |  | **Asian** | 994 | 6569 |  | **Std Deviation** | 11.84 | 12.68 |
| **Other** | 108 | 200 |  | **Black** | 3492 | 9160 |  |  |  |  |
|  |  |  |  | **Pacific Islander** | 85 | 327 |  |  |  |  |
|  |  |  |  | **White** | 23070 | 34648 |  |  |  |  |
|  |  |  |  | **Other** | 1591 | 4217 |  |  |  |  |
|  |  |  |  | **Unknown** | 13019 | 26523 |  |  |  |  |

| **Test Set 1** | | |  |  |  |  |  |  |  |  |
| --- | --- | --- | --- | --- | --- | --- | --- | --- | --- | --- |
| **Gender** | **Case** | **Control** |  | **Race** | **Case** | **Control** |  | **Age** | **Case** | **Control** |
| **Female** | 18388 | 26232 |  | **American Indian** | 165 | 289 |  | **Mean** | 55.87 | 55.24 |
| **Male** | 12803 | 35564 |  | **Asian** | 769 | 4970 |  | **Std Deviation** | 11.67 | 12.44 |
| **Other** | 60 | 108 |  | **Black** | 2505 | 6669 |  |  |  |  |
|  |  |  |  | **Pacific Islander** | 64 | 239 |  |  |  |  |
|  |  |  |  | **White** | 16783 | 26146 |  |  |  |  |
|  |  |  |  | **Other** | 1141 | 3088 |  |  |  |  |
|  |  |  |  | **Unknown** | 9824 | 20503 |  |  |  |  |

| **Test Set 2** | | |  |  |  |  |  |  |  |  |
| --- | --- | --- | --- | --- | --- | --- | --- | --- | --- | --- |
| **Gender** | **Case** | **Control** |  | **Race** | **Case** | **Control** |  | **Age** | **Case** | **Control** |
| **Female** | 1776 | 2916 |  | **American Indian** | 22 | 29 |  | **Mean** | 58.30 | 58.43 |
| **Male** | 1143 | 3642 |  | **Asian** | 49 | 335 |  | **Std Deviation** | 12.26 | 13.05 |
| **Other** | 0 | 0 |  | **Black** | 251 | 732 |  |  |  |  |
|  |  |  |  | **Pacific Islander** | 4 | 24 |  |  |  |  |
|  |  |  |  | **White** | 1503 | 2734 |  |  |  |  |
|  |  |  |  | **Other** | 87 | 262 |  |  |  |  |
|  |  |  |  | **Unknown** | 1003 | 2442 |  |  |  |  |

| **Test Set 3** | | |  |  |  |  |  |  |  |  |
| --- | --- | --- | --- | --- | --- | --- | --- | --- | --- | --- |
| **Gender** | **Case** | **Control** |  | **Race** | **Case** | **Control** |  | **Age** | **Case** | **Control** |
| **Female** | 1944 | 2294 |  | **American Indian** | 16 | 31 |  | **Mean** | 52.60 | 52.07 |
| **Male** | 1411 | 2975 |  | **Asian** | 39 | 213 |  | **Std Deviation** | 10.31 | 11.58 |
| **Other** | 3 | 6 |  | **Black** | 333 | 734 |  |  |  |  |
|  |  |  |  | **Pacific Islander** | 8 | 20 |  |  |  |  |
|  |  |  |  | **White** | 2004 | 2592 |  |  |  |  |
|  |  |  |  | **Other** | 133 | 298 |  |  |  |  |
|  |  |  |  | **Unknown** | 825 | 1387 |  |  |  |  |
